# *H19* is not hypomethylated or upregulated with age or sex in the aortic valves of mice

**DOI:** 10.14814/phy2.14244

**Published:** 2019-10-06

**Authors:** Mark Vander Roest, Christopher Krapp, Joanne L. Thorvaldsen, Marisa S. Bartolomei, W. David Merryman

**Affiliations:** ^1^ Biomedical Engineering Vanderbilt University Nashville Tennessee; ^2^ Epigenetics Institute Department of Cell and Developmental Biology University of Pennsylvania Perelman School of Medicine Philadelphia Pennsylvania

**Keywords:** *H19*, calcific aortic valve disease, age, epigenetics

## Abstract

Epigenetic dysregulation of long noncoding RNA *H19* was recently found to be associated with calcific aortic valve disease (CAVD) in humans by repressing *NOTCH1* transcription. This finding offers a possible epigenetic explanation for the abundance of cases of CAVD that are not explained by any clear genetic mutation. In this study, we examined the effect of age and sex on epigenetic dysregulation of *H19* and subsequent aortic stenosis. Cohorts of littermate, wild‐type C57BL/6 mice were studied at developmental ages analogous to human middle age through advanced age. Cardiac and aortic valve function were assessed with M‐mode echocardiography and pulsed wave Doppler ultrasound, respectively. Bisulfite sequencing was used to determine methylation‐based epigenetic regulation of *H19*, and RT‐PCR was used to determine changes in gene expression profiles. Male mice were found to have higher peak systolic velocities than females, with several of the oldest mice showing signs of early aortic stenosis. The imprinting control region of *H19* was not hypomethylated with age, and *H19* expression was lower in the aortic valves of older mice than in the youngest group. These results suggest that age‐related upregulation of *H19* is not observed in murine aortic valves and that other factors may initiate *H19*‐related CAVD in humans.

## Introduction

Calcific aortic valve disease (CAVD) is an increasingly prevalent source of cardiovascular morbidity in the elderly, but identifiable genetic causes only explain a small fraction of disease cases (Lindman et al. 2016; Stewart et al. 1997; Osnabrugge et al. 2013). *NOTCH1* loss of function mutation is one of the most widely studied genetic causes of CAVD, although such a mutation is not found in the majority of disease cases (Garg et al. 2005; Mohamed et al. 2006). Despite this discrepancy, alterations in the *NOTCH1* signaling pathway offer a proven mechanism for CAVD, and downstream mechanisms for valve calcification are similar for *Notch1*‐driven CAVD in mice and idiopathic CAVD in humans (Hutcheson et al. 2014; Chen et al. 2015; Clark et al. 2017). As a result, upstream signaling or alternative mechanisms to mimic *NOTCH1* loss of function have been sought as an explanation for idiopathic CAVD.

**Figure 1:**
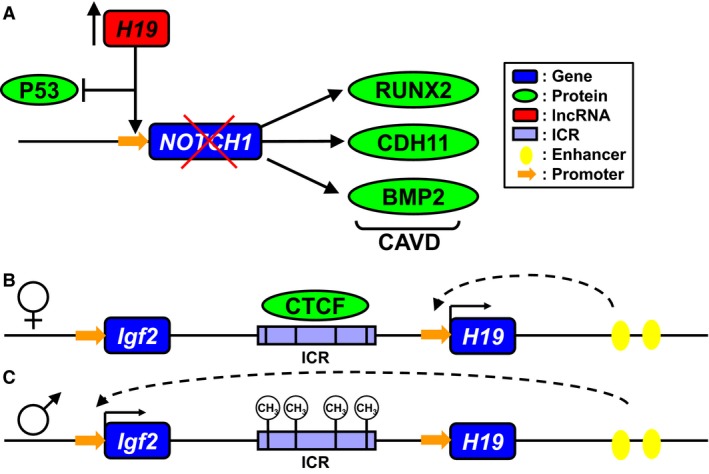
H19 imprinting and effect on CAVD. (A) High levels of *H19* compete with P53 to bind the NOTCH1 promoter, decreasing expression of *NOTCH1* and mimicking a loss‐of‐function mutation known to lead to CAVD. The *H19*/*IGF2* locus contains a differentially methylated domain in the intergenic space. On the maternally inherited allele (B) CTCF binds to a series of four 21bp repeats, resulting in interaction of downstream enhancers with the *H19* promoter and expression of *H19*. CTCF also acts as an insulator, keeping the downstream enhancers from promoting *Igf2* expression. On the paternal allele (C) methylation of the differentially methylated domain prevents CTCF from binding, enabling enhancer interaction with the *IGF2* promoter. *IGF2* expression is increased, while *H19* expression is almost entirely stopped. Hypomethylation in the ICR can lead to increased *H19* expression

Recently, long noncoding RNA *H19* has been found to be highly upregulated in stenotic and sclerotic human aortic valves (Hadji et al. 2016; Merryman and Clark 2016). Furthermore, *H19* was shown to competitively bind to the promoter region of *NOTCH1* in aortic valve interstitial cells, preventing P53 recruitment and subsequent *NOTCH1* transcription (Fig. 1A). This effectively suppresses *NOTCH1*, leading to calcification even in the absence of a *NOTCH1* mutation. However, this makes the means by which *H19* expression becomes so dramatically upregulated a critical question to understand disease initiation.

*H19* is highly conserved among mammals and is found in an imprinted locus near insulin‐like growth factor 2 (*Igf2*) (Bartolomei et al. 1991). This locus is epigenetically regulated by an imprinting control region (ICR), located between the two genes (Fig. 1)B and C) (Bartolomei et al. 1991). On the maternally inherited allele, the ICR binds CCCTC‐binding factor (CTCF), which serves as an insulator between *Igf2* and enhancer elements downstream of *H19*. As a result, shared enhancers promote expression of *H19* while *Igf2* is silenced (Fig. 1B). On the paternally inherited allele, methylation of the ICR prevents CTCF binding, allowing the downstream enhancers to activate *Igf2* expression while *H19* is silenced (Fig. 1C) (Engel et al. 2008). This imprint is established during embryonic development and persists through the life of the organism, though disruptions in this epigenetic signature could lead to rapid changes in *H19* expression (Gabory et al. 2006).

Hadji et al. showed that hypomethylation in the promoter region of *H19* was associated with increased expression in calcified human aortic valves, even though the imprint was maintained (Hadji et al. 2016). More recently, Agba et al. showed evidence for age‐associated hypomethylation in the *H19*/*Igf2* ICR of rats, suggesting a loss of imprint which correlated with increased *H19* expression (Agba et al. 2017). Together, these findings suggest an epigenetic mechanism by which *H19* may become upregulated with advanced age and lead to CAVD via the *NOTCH1* pathway, even in the absence of a genetic mutation.

The goal of this study was to determine if these findings were replicated in mouse aortic valves, and if age‐related *H19* expression is a mechanism for CAVD in a mouse model. Because *H19* is known to be involved in other cardiovascular diseases, expression levels were also assessed in ventricular tissue and the aortic arch, as well as the liver, which is known to express higher levels of *H19* and was previously shown to exhibit age‐related loss of imprint. While many existing mouse models of CAVD consider only male mice, CAVD is also highly prevalent in women, thus motivating our investigation into the effect of sex on *H19*‐driven calcification (Owens et al. 2010; Simard et al. 2017). Because our primary interest was in the effects of age and sex (rather than genetic mutation or chronic injury through diet) on *H19* expression in the aortic valve, these experiments were conducted in healthy C57BL/6 mice maintained on a normal diet.

## Methods

### Mice

Groups of five male and female C57BL/6 mice were purchased from Jackson Laboratory at 26, 52, and 78 weeks of age. Mice were assessed via echocardiography and euthanized for sample collection within two weeks of receipt. All procedures were performed in accordance with protocols approved by Institutional Animal Care and Use Committee (IACUC) at Vanderbilt University.

### Echocardiography

**Figure 2:**
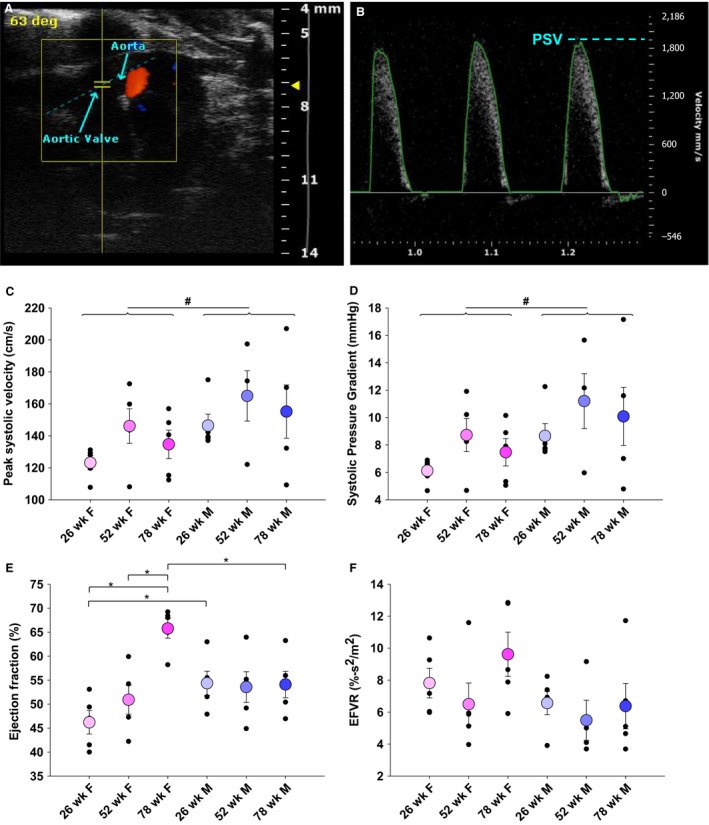
Valve health in male mice diverges with age. Pulsed wave Doppler ultrasound of aortic flow immediately distal to the aortic valve (A) with representative flow profile (B). Echocardiography found elevated PSV and transvalvular pressure gradient in male mice (*P* = 0.032, *P* = 0.032) and trends of higher and more divergent velocities and pressure gradient with increasing age in male mice (C and D). Ejection fraction was consistent for males, but increased with age in females (E). EFVR showed similar trends as PSV and pressure gradient (F). *P < 0.05 between individual groups by two‐way ANOVA; ^#^ *P* < 0.05 between sex by two‐way ANOVA; PSV: peak systolic velocity; EFVR: ejection fraction to velocity ratio

Mice were anesthetized with isofluorane, and a Vevo 2100 imaging system was used to acquire parasternal, short axis M‐mode images of the heart and pulsed‐wave (PW) Doppler images of the aorta immediately distal to the aortic valve. An exemplary PW Doppler scan and corresponding flow profile traces are shown in Figure 2A and B. VevoLAB software was used to analyze M‐mode cardiac cycles (~9 per image) and extract PW Doppler images. A custom MATLAB script was used to isolate and average PW Doppler cardiac cycles (~10–30 per image) in order to compute the systolic transvalvular pressure gradient and peak systolic velocity (PSV) (Ferruzzi et al. 2018). Ejection fraction to velocity ratio (EFVR), a metric used as an indicator of valve disease, was calculated as EFVR = (ejection fraction)/(4*(PSV)^2^) (Cattaneo et al. 2009).

### Microdissection and sample collection

Mice were euthanized by carbon dioxide inhalation and promptly dissected. The systemic and pulmonary circulatory systems were flushed with sterile PBS, and the aortic valve leaflets – connected to a minimal annulus of aorta – were dissected away from the ventricles. Samples of liver, left ventricle, and ascending aorta were also harvested and cleaned of external connective tissue and fat. Samples were flash frozen in liquid nitrogen and stored at −80°C until RT‐PCR analysis.

### Nucleic acid purification

Tissue samples were thawed at room temperature and bead homogenized in 400 *μ*L of RLT‐plus buffer with Reagent DX to reduce foaming (Qiagen, Hilden, Germany) in Lysing Matrix D tubes (MP Biomedicals, Santa Ana, CA) until no visible tissue remained. RNA and gDNA were purified using the Qiagen AllPrep Micro kit following manufacturer’s instructions and were stored at −80°C until further analysis.

### Real‐time PCR

Reverse transcription was performed using the SuperScript IV Reverse Transcriptase kit with oligo(dT) primer (ThermoFisher Scientific, Waltham, MA). RT‐PCR was performed with equal amounts of cDNA using iQ SYBR Green Supermix (Bio‐Rad, Hercules, CA) and gene specific primer sequences (Table 1). Gene expression was normalized to the geometric mean expression of *Gapdh*, *Tuba1b*, and *Actb*, which was found to be more stable than any housekeeping gene in isolation. For visual clarity, gene expression was normalized to the highest expressing sample of each gene.

**Table 1:** RT‐PCR primer sequences

Gene name	Forward primer^1^	Reverse primer^1^
*Gapdh*	ATGACAATGAATACGGCTACAG	TCTCTTGCTCAGTGTCCTTG
*Tuba1b*	CCGGTGTCTGCTTCTATCTC	CCATGTTCCAGGCAGTAGAG
*Actb*	CAAGCAGGAGTACGATGAGTC	AACGCAGCTCAGTAACAGTC
*H19*	GGAATGTTGAAGGACTGAGGG	GTAACCGGGATGAATGTCTGG
*Igf2*	CGCTTCAGTTTGTCTGTTCG	GCAGCACTCTTCCACGATG
*Notch1*	ATGTCAATGTTCGAGGACCAG	TCACTGTTGCCTGTCTCAAG
*Bmp2*	TTATCAGGACATGGTTGTGGAG	GGGAAATATTAAAGTGTCAGCTGG

^1^ Primer sequences are from 5′‐3′.

### Methylation analysis

Pyrosequencing was performed as previously described with the following modifications. 40 ng of bisulfite treated DNA was used as input, and 5 *μ*L of the biotinylated PCR product was used for each sequencing assay (Susiarjo et al. 2013).

### Statistics

All values are presented as mean ± standard error. Two‐way ANOVA with post hoc Holm‐Sidak test for multiple comparisons was used to detect differences between age and sex. In the event that conditions of normality or equal variance were not met, one‐way ANOVA on ranks was used to detect differences due to age within each sex, and the Mann‐Whitney rank sum test was used to detect difference due to sex at a specific age. Potential correlations between measured variables were assessed by the Pearson product‐moment correlation r. For all statistical tests, a value of *P* < 0.05 was considered significant.

## Results

### Aortic valve health diverges, but does not worsen with age

Analysis of PW Doppler images revealed no changes with age in PSV or peak transvalvular pressure gradient, although as whole cohorts by sex, male mice had higher PSV than females (*P* = 0.032) (Fig. 2C and D). While typical values for aortic PSV in healthy BL6 mice fall below 150 cm/sec, we identified one 78‐week‐old male mouse had a PSV over 200 cm/sec, indicative of aortic stenosis, and several older male and female mice had velocities between 150 and 200 cm/sec, indicative of early stenosis (Hinton et al. 2008). Male mice also had higher systolic gradients (*P* = 0.032). None of the mice showed signs of left ventricular hypertrophy, indicating that observed hemodynamic changes were early stage, prior to extensive cardiac remodeling. Curiously, the 52‐ and 78‐week‐old male mice had two and three times larger variance in PSV than younger males, indicating a wide divergence of overall valve health with increased age.

### Cardiac function is preserved with age

Ejection fraction was extremely consistent with age in male mice (Fig. 2E). Female mice exhibited increasing ejection fraction with age, such that 26‐week‐old females had lower ejection fraction than 26‐week‐old male mice (*P* = 0.042) and 78‐week‐old female mice had higher ejection fractions than 78‐week‐old males (*P* = 0.005). Seventy‐eight‐week‐old females also had higher ejection fractions than 26‐ and 52‐week‐old females (*P* = 0.017, *P* = 0.025). No differences were detected in EFVR, although males again showed trends toward divergent valve health with age (Fig. 2F).

### H19 imprint and expression are not altered by age or sex in the aortic valve

**Figure 3:**
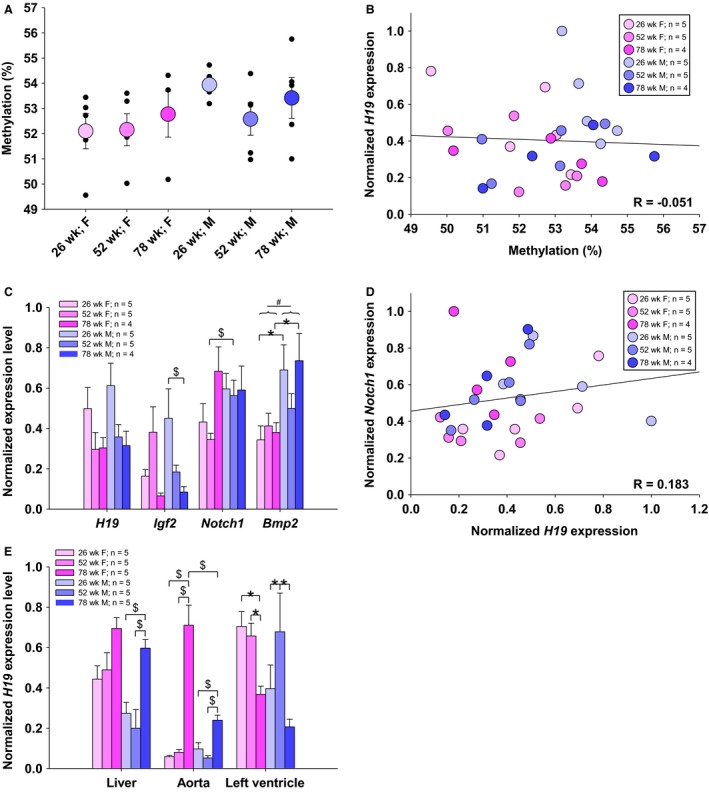
Methylation and expression of *H19* and downstream genes is largely unchanged with age and sex. *H19* ICR methylation was unchanged in all samples studied (A) and did not correlate with *H19* expression (B). Expression of *H19* was significantly higher in 26‐week‐old mice than in 52‐week‐old mice (*P* = 0.011) and 78‐week‐old mice (*P* = 0.012), but did not correlate with *Notch1* expression (C, D). *Igf2* and *Notch1* were found to be differ in individual comparisons, and *Bmp2* was higher in males than in females (*P* = 0.002). *H19* expression was higher at 78 weeks in the liver and aorta of male mice and in the aortas of female mice. No clear trend in *H19* expression was found in the left ventricle, although 78‐week‐old female mice had lower expression (*P* = 0.005, 78 week to 26 week; *P* = 0.014, 78 week to 52 week) and 52‐week‐old males had higher expression (*P* < 0.001, 52 week to 26 week; *P* < 0.001, 52 week to 78 week) than other aged mice of the same sex. *P < 0.05 between individual groups by two‐way ANOVA; ^$^ *P* < 0.05 between individual groups by ANOVA on ranks or Mann‐Whitney; ^#^ *P* < 0.05 between sex by two‐way ANOVA

Pyrosequencing of the *H19* ICR in mouse aortic valve gDNA revealed no change in average methylation fraction due to age or sex (Fig. 3A). Likewise, there was no observed trend toward increased *H19* expression in the valve with increasing age nor correlation between methylation fraction and *H19* expression (Fig. 3B and C). Rather, 26‐week‐old mice had higher average *H19* expression than either the 52‐ or 78‐week‐old mice (*P* = 0.025; *P* = 0.017). Expression of *Igf2*, which is regulated by the *H19/Igf2* ICR, and *Notch1*, which was previously shown to be repressed by *H19*, also showed no clear age or gender effect, though individual comparisons reached statistical significance (Fig. 3C). *Bmp2*, a driver of osteogenic calcification that has been found to be increased in calcified valves, was higher in male mice than in females overall (*P* = 0.002) and specifically at 26 and 78 weeks of age (*P* = 0.015; *P* = 0.017). To examine if *H19* may still be suppressing *Notch1* on an individual level that is not revealed in group‐averaged data, we also correlated expression of *H19* and *Notch1* in individual mice, but no significant effect of *H19* expression on *Notch1* expression was detected (Fig. 3D).

### Liver and aortic tissue show increased H19 expression in oldest mice

Based on other studies showing *H19* upregulation in aortic aneurysm and cardiac ischemia, as well as a study that found age‐related loss of imprint in rat liver, we also probed *H19* expression changes in the liver, left ventricle, and ascending aorta (Greco et al. 2016; Agba et al. 2017; Li et al. 2018) (Fig. 3E). The 78‐week‐old male mice had higher *H19* expression than other age males in both the liver and ascending aorta. *H19* expression trended upwards with age in female liver, while the ascending aorta of 78‐week‐old female mice showed higher expression than the other female age groups and the 78‐week‐old male mice. In left ventricular tissue, no clear pattern of age‐related *H19* upregulation was observed, though the 78‐week‐old female mice had significantly lower *H19* expression than other female age groups, and 52‐week‐old males had higher expression than other male age groups.

## Discussion

This study investigated the effects of age and sex on hemodynamic function and *H19* expression in the aortic valve. To the best of our knowledge, this is the first cross‐sectional study of a potential CAVD‐initiating mechanism in both male and female mice that did not have a CAVD‐associated mutation or receive a chronic hypercholesteremic treatment. Furthermore, this study isolated nucleic acids from individual aortic valves, enabling expression profiling and methylation analysis without requiring sample pooling. This approach represents a novel approach to probe subtle signaling which may precede or initiate valve disease in mice without introducing the biases of specific models, whereas most studies (including our own (Clark et al. 2017)) typically utilize specific mutations, diet, or cardiovascular injury to induce symptoms of CAVD, which may bias or obscure subtle signaling changes early in disease progression.

We found that the *H19*/*Igf2* ICR does not undergo age‐related hypomethylation and *H19* is not upregulated in mouse aortic valves with age alone. This contrasts with expression data from other tissues such as liver and ascending aorta, as well as with prior studies in rats that showed loss of ICR methylation and *H19* upregulation in a tissues such as brain and skin (Agba et al. 2017). The rats used in that study and the mice used here included animals comparable to 60‐ to 70‐year‐old humans, well within a timeframe at which early signs of CAVD could be expected. These results suggest a mechanism for preserving *H19* imprinting and low expression levels that differs by tissue type or species.

In addition to the lack of a robust increase in *H19* expression, we did not observe substantial correlation between expression of *Notch1* and *H19* in the aortic valve. Previous studies have shown that elevated *H19* can repress *NOTCH1* in human aortic valve interstitial cells and mouse brain tissue (Hadji et al. 2016). A potential explanation for this discrepancy is a threshold effect, in which *H19* expression must reach a certain critical level before measurably repressing *Notch1*. Further work with a titratable *H19* overexpression system may be a useful tool to answer this question.

Despite the lack of strong *H19* upregulation, a few of the oldest mice showed signs of early aortic stenosis as indicated by elevated PSV and transvalvular pressure gradient. These functional indicators of stenosis even in the absence of *H19* upregulation underscore the heterogeneous nature of CAVD and suggest that *H19* is not the sole initiator of disease. Nearly 30 different mouse models of CAVD exist, and while many of these models are convergent, it is clear that multiple distinct mechanism can initiate and drive CAVD (Sider et al. 2011; Hutcheson et al. 2014; Lerman et al. 2015).

Certain sex‐related differences emerged in this study which corroborate known statistics of human disease. For example, male mice had higher overall PSV values that trended upwards with age and higher *Bmp2* expression, indicative of onset of stenosis with activation of calcific pathways. This matches with clinical data showing that men are more likely to develop CAVD than women and that features of the disease (fibrosis and calcification) differ between sexes (Owens et al. 2010; Simard et al. 2017). Still, CAVD is relatively prevalent in women, and the differences in disease progression between sexes may direct more personalized treatment strategies. Despite this, many studies either do not distinguish between male and female mice or use only males. The work presented here included both male and female mice in order to better capture any differences in *H19* regulation and valve stenosis.

Although age‐related upregulation of *H19* was not found in the aortic valve, it was confirmed in the liver and reported for the first time in the ascending aorta. It was also not found to increase in the left ventricle, showing a high degree of tissue specificity in overall expression regulation. Many studies have identified *H19* as a biomarker or driver of other cardiovascular diseases such as aortic aneurysm, smooth muscle cell apoptosis, endothelial cell aging, and ischemic heart failure (Greco et al. 2016; Li et al. 2018; Hofmann et al. 2019). Our findings of tissue‐specific differences in age‐related upregulation of *H19* may inform future work looking at disease initiating events in these other pathologies.

This study is not without its limitations. One of the biggest challenges was the sample size – both of individual aortic valves and the overall size of the cohort. To obtain testable quantities of RNA and DNA, valves were used entirely for nucleic acid extraction. Alternate methodologies such as histology, immunohistochemistry, or *in situ* hybridization may have revealed more information about the extent of valve remodeling and stenosis. Furthermore, the high cost of raising or purchasing aged mice prevented higher numbers in each cohort, which may have increased the statistical power and significance of these results. Finally, the methylation sites tested in this study were within the ICR, which is typically considered to be the site of most epigenetic regulation of *H19* expression and was shown to be hypomethylated with age in work by Agba et al. (2017). However, Hadji et al. report a stronger association of *H19* expression with a particular CpG site in the promoter region of *H19* (Hadji et al. 2016). Despite this difference, we did not observe the increase in *H19* expression that was clearly reported in their work.

This work shows a lack of age‐related epigenetic dysregulation of *H19* in mouse aortic valves, even while other tissue types demonstrated consistent upregulation of *H19* expression. Nevertheless, echocardiographic metrics and changes in gene expression in individual mice showed signs of valve remodeling and early stenosis. Together, these results show that *H19* loss of imprint and subsequent upregulation are not common in the aortic valve of old mice, and that increased *H19* levels do not appear to be a prerequisite for early stage valve disease.

## Conflict of Interest

No conflicts of interest, financial or otherwise, are declared by the authors.
